# Busulfan inhibits *Pseudomonas aeruginosa* growth and reduces biofilm biomass and pyocyanin production

**DOI:** 10.3389/fcimb.2025.1721773

**Published:** 2026-01-02

**Authors:** Needa A. Bahkali, Rhodanne Nicole A. Lambarte, Terrence S. Sumague, Turki M. Dawoud, Khalid S. Almaary, Abdurahman A. Niazy

**Affiliations:** 1 Department of Botany and Microbiology, College of Science, King Saud University, Riyadh, Saudi Arabia; 2 Molecular and Cell Biology Laboratory, Prince Naif bin AbdulAziz Health Research Center, King Saud University Medical City, Riyadh, Saudi Arabia; 3 Department of Oral Medicine and Diagnostic Sciences, College of Dentistry, King Saud University, Riyadh, Saudi Arabia

**Keywords:** busulfan, anticancer, drug repurposing, *Pseudomonas aeruginosa*, virulence factors, antibacterial

## Abstract

**Background:**

*Pseudomonas aeruginosa* remains a serious threat in clinical settings, especially among patients who are immunocompromised, receiving chemotherapy, or in intensive care units. With the rise of antibiotic resistance, drug repurposing offers a promising alternative strategy. Busulfan, an anticancer alkylating agent that induces DNA cross-linking and cytotoxic effects in cancer cells, may exert similar effects on microorganisms, as reported for other alkylating agents.

**Methods:**

This study evaluated the antibacterial potential of busulfan against *P. aeruginosa*. Initially, the antimicrobial activity of busulfan was assessed using the microdilution method, followed by investigations of key virulence factors of the PAO1 strain after treatment.

**Results:**

Busulfan inhibited bacterial growth in a dose-dependent manner, with 84% inhibition observed at 108 μg/mL, whereas bactericidal activity was only observed at much higher concentrations (MBC >512 and <1,024 μg/mL). Busulfan significantly reduced biofilm formation by 55%, decreased live-cell viability by 67% as observed using confocal laser scanning microscopy (CLSM), decreased pyocyanin production by 57%, and impaired iron chelation by 25%. Moreover, moderate synergy with gentamicin was observed at higher concentrations of busulfan. However, treatment with 108 μg/mL busulfan showed no effect on PAO1 hemolysis or motility.

**Conclusion:**

Overall, busulfan demonstrates antimicrobial activity against *P. aeruginosa*, particularly through its effects on virulence factors. These preliminary results support the potential value of busulfan for repurposing, although further studies are needed to clarify its mechanism and therapeutic relevance.

## 1 Introduction

*Pseudomonas aeruginosa* is a gram-negative, rod-shaped, opportunistic pathogen known for its ability to acquire multidrug resistance (MDR), which contributes to its environmental ubiquity, including in healthcare settings, making it a life-threatening organism for hospitalized patients, especially those in intensive care units (Wu et al., 2015). It is a threat to individuals recovering from chemotherapy or organ transplantation, those who are immunocompromised due to cancer, and patients with cystic fibrosis (Botzenhart and Doring, 1993; Silva et al., 2020; Kang et al., 2021). According to Lister et al., leukemia and immunocompromised patients are more vulnerable to infection with MDR *P. aeruginosa* due to the use of invasive devices (Lister et al., 2009; Di Domenico et al., 2022). A 2025 BMC Infectious Diseases study reported 52 P*. aeruginosa* bloodstream isolates collected from hospitalized cancer patients, underscoring the continued prevalence of the pathogen in this vulnerable population (Abdulhak et al., 2025). An earlier study indicated that the prevalence of *P. aeruginosa* among isolates from cancer patients was 54% (Ahmed et al., 2020). It is also a common source of bacteremia in catheterized patients and in serious burn victims (Stover et al., 2000). During the coronavirus pandemic, *P. aeruginosa* was identified as one of the most prevalent bacterial pathogens in COVID-19 patients (Chandrasekaran and Fernandes, 2020).

The ESKAPE group (*Enterococcus faecium, Staphylococcus aureus, Klebsiella pneumoniae, Acinetobacter baumannii, Pseudomonas aeruginosa*, and *Enterobacter* spp.) has long been recognized as a major cluster of MDR pathogens responsible for most nosocomial infections. According to the updated WHO Bacterial Priority Pathogens List, carbapenem-resistant *P. aeruginosa* has recently been reclassified from the “critical” to the “high priority” group. Despite this revision, *P. aeruginosa* remains a clinically important pathogen with substantial virulence and biofilm-associated challenges (Sati et al., 2024). The accelerating emergence of antibiotic resistance, coupled with the limited pace of new antibiotic development, has prompted increased interest in repurposing approved drugs for antimicrobial use (Di Bonaventura et al., 2023). This approach of re-evaluating existing compounds for new therapeutic applications presents several benefits over conventional drug discovery, with the potential to save significant time and resources (Barbarossa et al., 2022).

Although anticancer agents are known for their systemic toxicity at therapeutic doses, drug-repurposing research has further investigated them as potential molecular probes that can reveal vulnerabilities in bacterial physiology or serve as scaffolds for developing safer derivatives. Several anticancer compounds, including 5-fluorouracil, mitomycin C, and cisplatin, have shown antimicrobial or antivirulence activity at concentrations significantly lower than clinically cytotoxic levels (Niazy et al., 2024a; Svedholm et al., 2024; Daghistani et al., 2025). These findings support the broader idea that drugs targeting DNA integrity, nucleotide synthesis, or redox balance in cancer cells may also interfere with bacterial growth or virulence pathways, providing a rationale for studying the effects of busulfan (Bu) on *P. aeruginosa.*

Although bacteria and cancer cells differ fundamentally, they share certain vulnerable cellular features, such as susceptibility to disturbances in DNA replication, metabolic stress, and redox balance (Huang and Zhou, 2021). These shared susceptibilities have motivated the repurposing of several anticancer agents as antimicrobials. For example, drugs that interfere with nucleotide synthesis, DNA integrity, or cellular redox homeostasis can affect rapidly dividing cancer cells and metabolically active bacterial pathogens (Li et al., 2023). Furthermore, bacteria frequently develop biofilms that mimic solid tumors due to the complexity of their microenvironment and cellular states (Soo et al., 2017; Quezada et al., 2020). Several virulence factors utilized by *P. aeruginosa* play crucial roles in its pathogenesis, such as biofilm formation, quorum-sensing regulatory proteins, pigment production, flagella and pili, elastase, and exotoxins.

**Figure 1:**
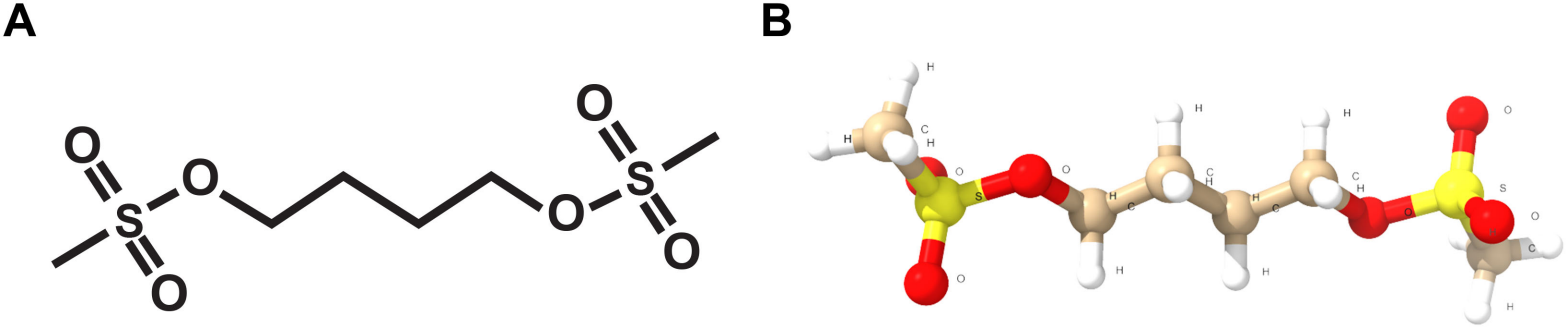
**(A)** Two-dimensional and **(B)** three-dimensional chemical structures of busulfan.

Busulfan (**Figure 1**) is a bifunctional alkylating agent belonging to the alkyl sulfonate class of chemotherapeutics. It undergoes a nucleophilic substitution (SN2) reaction with guanine bases in DNA, resulting in the formation of intra- or interstrand cross-links. The *in vitro* cytotoxicity of Bu is associated with the accumulation of DNA lesions. It also promotes the formation of DNA–protein cross-links by interacting with cysteine molecules on histone proteins. Bu’s alkylating activity disrupts cellular processes such as DNA replication, damage-repair mechanisms, and gene transcription (Myers et al., 2017). Bu has demonstrated mutagenic effects in microorganisms, cultured mammalian cells, rodent models, and Drosophila (Bishop and Wassom, 1986). Bu’s DNA-alkylating and cross-linking activities, along with its redox-imbalancing effects, align with the known antibacterial mechanisms of compounds such as mitomycin C. These mechanisms(—DNA damage and oxidative stress——)are well-established triggers for bacterial SOS and stress responses, supporting the idea that Bu could exert antibacterial effects in *P. aeruginosa* (Fan and Peng, 2016).

Moreover, combination therapy offers a valuable approach for improving treatment effectiveness against infections caused by resistant bacteria. Drug interactions can be assessed using techniques such as the checkerboard assay, which provides an *in vitro* evaluation of potential synergistic or antagonistic effects. Given the increasing incidence of antibiotic-resistant *P. aeruginosa*, examining combination regimens through such assays is essential for identifying improved therapeutic strategies (Black et al., 2023). In this study, gentamicin and ciprofloxacin were selected because they target distinct essential pathways-protein synthesis via the 30S ribosomal subunit and DNA gyrase/topoisomerase II, respectively, providing mechanistic complementarity to Bu’s DNA-alkylating effects (Ruden et al., 2019). This rationale aligns with prior findings showing that agents acting on different bacterial processes can potentiate each other. Ruden et al. demonstrated that several short cationic antimicrobial peptides exhibited strong synergy with ribosome-targeting antibiotics and, to a lesser extent, with ciprofloxacin, with some combinations achieving FIC values ≤0.5 against MDR *P. aeruginosa* strains (Ruden et al., 2019). Together, these observations support evaluating whether Bu could similarly enhance antibiotic activity or attenuate virulence at sub-inhibitory concentrations.

Furthermore, molecular docking is one of the cornerstone approaches in drug-repurposing studies, as it predicts the interaction and binding stability of existing compounds with known target proteins and accelerates the identification of potential candidate compounds for drug development (Glajzner et al., 2024). PAO1 pathogenicity is commonly governed by known genes associated with pigment production and biofilm formation; among these are lasR and pqsE, which regulate motility and siderophore production; qscR, which modulates quorum sensing; and alg44, which influences biofilm structure and motility (Ding et al., 2018; Díaz-Pérez et al., 2023). Therefore, selecting these genes to correlate drug interactions with observed phenotypic assays provides a rational approach and mechanistic insight for drug-discovery strategies.

*P. aeruginosa* PAO1 is widely used as a standard laboratory reference strain and serves as a model organism for biofilm research due to its fully sequenced genome, which is available in public databases (Stover et al., 2000; Grace et al., 2022).

This study aimed to investigate the potential of repurposing the anticancer drug Bu for the treatment of *P. aeruginosa in vitro*. We focused on testing the inhibitory effects of Bu on several virulence factors utilized by *P. aeruginosa* that play crucial roles in pathogenesis.

## 2 Materials and methods

### 2.1 Bacterial strain and growth media

The strain used in this study was the wild-type (WT) *P. aeruginosa* PAO1, a kind contribution from Dr. Lee Hughes at the University of North Texas, Texas, USA (Brichta et al., 2004; Niazy et al., 2022). The strain was preserved at −80°C in tryptic soy broth (SPML Co. Ltd., Riyadh, Saudi Arabia) supplemented with 10% glycerol (Sigma-Aldrich, St. Louis, MO, USA).

For each experiment, tryptic soy agar (SPML) was used to freshly subculture the PAO1 strain, which was incubated overnight at 37 °C. A single isolated colony was then inoculated into 10 mL of *Pseudomonas* minimal medium (PsMM), prepared according to the method described by Ornston and Stanier (1966), with 20% glucose added as the carbon source. The culture was incubated overnight at 37 °C with shaking at 150 rpm using an Excella E24 incubator shaker (New Brunswick Scientific, Edison, NJ, USA). The bacterial suspension was considered ready for subsequent experimental use when it reached mid-log phase (OD_600_ between 0.4 and 0.6), as measured using a spectrophotometer (Libra S22, Biochrom Ltd., Cambridge, UK). All experiments were performed using three biologically independent replicates in triplicate.

### 2.2 Anticancer drug and antibiotics

The anticancer agent Bu (Pharmascience Inc., Quebec, Canada) was selected as the cytotoxic model drug for this study. Bu suspensions were prepared in sterile saline (Pharmaceutical Solutions Industry, Jeddah, Saudi Arabia) and used immediately due to their limited stability (Guichard et al., 2017).

Throughout the study, the direct dilution method was used to prevent cross-contamination (Olechno et al., 2013). Bu was considered inhibitory when it resulted in at least 80% growth inhibition based on optical density (OD), according to the following formula: % inhibition = ((OD_(untreated)_ − OD_(test)_)/OD_(untreated)_) × 100 (Koshelev et al., 2020).

Gentamicin (30S ribosome inhibitor) (Gibco by Life Technologies, Grand Island, NY, USA) and ciprofloxacin (DNA gyrase/topoisomerase II inhibitor) (Quinox, Tabuk Pharmaceuticals, Tabuk, Saudi Arabia) were selected for the checkerboard assay due to their complementary mechanisms and routine clinical use against *P. aeruginosa*. The susceptibility of planktonic cells was evaluated using EUCAST v14.0 breakpoints (e.g., ciprofloxacin S ≤0.25/R >0.5 mg/L), while biofilm responses were measured through experimental indicators such asas biomass reduction and metabolic activity. The powdered forms of the drugs were dissolved in distilled water to prepare stock solutions (Weinstein and Lewis, 2020; Committee et al., 2024).

### 2.3 Minimum inhibitory concentration and minimal bactericidal concentration determination

The MIC of Bu was determined using the broth microdilution method, following Clinical and Laboratory Standards Institute (CLSI) guidelines with slight modifications (Balouiri et al., 2016). A series of two-fold serial dilutions of Bu (ranging from 1 to 2,048 µg/mL) was prepared to evaluate its inhibitory effects on the growth of PAO1 (Kowalska-Krochmal and Dudek-Wicher, 2021; Yan et al., 2023). For subsequent experiments, four concentrations (0.5, 3, 18, and 108 µg/mL) were selected based on a six-fold dilution series starting from 0.5 µg/mL, as these were within the range at which Bu achieved at least 80% growth inhibition (Koshelev et al., 2020; Bellio et al., 2021). In brief, 96-well microtiter plates (Greiner Bio-One GmbH, Frickenhausen, Germany) were prepared containing drug solutions, PsMM, and bacterial suspension. The plates were then incubated at 37 °C for 18–20 h. The positive control was untreated bacterial suspension, while the negative control consisted of the highest concentration of Bu without bacteria. The optical density at 600 nm (OD_600_) was measured using a BioTek Synergy HT microplate reader (BioTek Instruments, Winooski, VT, USA) to assess bacterial growth. Growth inhibition at each concentration was calculated relative to the untreated control after subtracting the baseline OD (0 h) and expressed as a percentage (Kowalska-Krochmal and Dudek-Wicher, 2021).

The MBC of Bu against the PAO1 strain was confirmed by transferring 10 µL from all wells of the MIC assay and from concentrations where bacterial growth was inhibited by >80% onto pre-prepared PsMM agar plates. The plates were spread evenly and incubated for 18–20 h at 37 °C (Hyatt et al., 1994). The MBC was defined as the lowest concentration of Bu at which no visible bacterial growth was observed.

### 2.4 Growth curve

*The P. aeruginosa* PAO1 strain was grown in PsMM in the presence of Bu to evaluate its effect on bacterial growth. The bacterial culture was first grown to the early exponential (log) phase, with an OD_600_ of approximately 0.2. PsMM and Bu concentrations (total volume of 95 µL) were added to the wells of 96-well microtiter plates, followed by the addition of a 5 µL aliquot of standardized bacterial culture to each well. Positive controls consisted of bacterial cultures without Bu treatment, while negative controls contained only the highest Bu concentration without bacteria. Cell density was measured at 600 nm every 15 min and recorded hourly for 18–20 h (until the stationary phase was reached) using the BioTek Synergy HT microplate reader (BioTek Instruments, Winooski, VT, USA) (Niazy et al., 2024b).

### 2.5 Biofilm assays

#### 2.5.1 Biofilm formation

The effect of Bu on biofilm formation of PAO1 was assessed using 96-well microtiter plates. Each well contained PsMM, the bacterial suspension, and Bu concentrations, as previously described. After 48 h of static incubation, the media and nonadherent bacteria were discarded by washing the plates carefully three times with distilled water. Then, 150 µL of 0.5% crystal violet solution (Sigma-Aldrich) was added to each well, left at RT for 15 min, and rinsed twice with distilled water to remove excess dye. Next, 200 µL of 95% ethanol (Sigma-Aldrich) was added, followed by incubation for 15 min. Each well was mixed thoroughly by pipetting to ensure a homogeneous solution. The absorbance was measured at 490 nm using a BioTek Synergy HT microplate reader. Biofilm formation was expressed as a percentage (Niazy et al., 2024a; Niazy et al., 2024b).

#### 2.5.2 Resazurin biofilm quantification assay

The resazurin assay was performed to quantify the effect of Bu on the metabolic activity of PAO1 biofilm. Biofilms were grown in 96-well microtiter plates containing PsMM, bacterial suspension, and Bu concentrations, as previously described, for 48 h under static conditions. The plates were then washed three times with phosphate-buffered saline (PBS) to remove the media and nonadherent bacteria. Next, 100 µL of saline was added to each well, mixed by pipetting to detach biofilm cells, and followed by the addition of 20 µL of resazurin solution (Sigma-Aldrich, St. Louis, MO, USA). The plates were incubated in the dark for 1 h at 37°C, and fluorescence was measured (λ_ex_: 560 nm and λ_em_: 590 nm) using a BioTek Synergy HT microplate reader (BioTek Instruments, Winooski, VT, USA) (Peeters et al., 2008; De Bleeckere et al., 2023).

#### 2.5.3 Live/dead assay

The effect of Bu on PAO1 biofilms was evaluated using confocal laser scanning microscopy (CLSM) (Nikon C2, Nikon Instruments Inc., Tokyo, Japan). PAO1 was cultured on coverslips in 6-well culture plates containing Bu concentrations and PsMM and incubated for 48 h at 37 °C. After incubation, the remaining broth was removed and PBS was used three times to wash and remove excess planktonic cells. The coverslips were gently transferred to a new 6-well plate, and 200 µL of LIVE/DEAD™ BacLight™ working solution (Invitrogen Ltd., Paisley, UK) was added to each coverslip and incubated at RT in the dark for 30 min. The morphology of the PAO1 biofilms that developed with and without Bu was studied using CLSM. The excitation/emission wavelengths were 488 nm/<550 nm for SYTO^®^ 9 (BacLight™ Component A) and 568 nm/>600 nm for propidium iodide (BacLight™ Component B). Images of the biofilm samples were acquired and rendered into three-dimensional plots using NIS-Elements Advanced Research Software (version 4.0, Nikon, Tokyo, Japan). Subsequent image analysis was performed with ImageJ software (version 1.50i, NIH, Bethesda, MD, USA). The inhibitory effect on biofilm depth and formation after treatment with Bu concentrations was visualized and evaluated on the generated optical slides (Niazy et al., 2024a).

### 2.6 Toxin production

The hemolysis assay was performed using 5% sheep blood agar plates (SAMCO, Riyadh, Saudi Arabia). One milliliter of bacterial suspension from the PAO1 strain was transferred into ten 1.5 mL Eppendorf tubes, followed by centrifugation at 10,100 × g for 2 min using an Eppendorf 5418 centrifuge (Hamburg, Germany). After discarding the supernatant, the cell pellets were retained and resuspended in 50 µL of PsMM and Bu (0.5, 3, 18, and 108 µg/mL). Each tube was vortexed to ensure proper mixing. Then, 5 µL of each resuspended sample was spotted onto the surface of blood agar plates and incubated at 37 °C for 20 h. Images were captured using a Nikon D3100 DSLR camera (Niazy and Hughes, 2015).

### 2.7 Pigments production

#### 2.7.1 Pyocyanin production assay

The inhibitory effect of Bu on the production of the bluish-green pigment pyocyanin by *P. aeruginosa* was assessed using King’s A broth. The method was adapted from a previous study (Fekete-Kertész et al., 2024) with slight modifications. King’s A medium was prepared according to the protocol of King, Ward, and Raney (King et al., 1954). The bacterial strain was cultured in King’s A broth containing different concentrations of Bu for 20 h at 37°C with shaking at 150 rpm. The absorbance of pyocyanin production was measured at 600 nm using a BioTek Synergy HT microplate reader. Subsequently, 5 mL of each culture was transferred to 15 mL conical tubes and centrifuged at 3,000 rpm for 25 min using a HERMLE Z 206 A centrifuge (Wehingen, Germany). The supernatant was then filtered through a 13 mm, 0.45 μm PES syringe filter (Hyundai Micro Co. Ltd., Seoul, South Korea) into new 15 mL conical tubes. Next, 3 mL of chloroform (Sigma-Aldrich, St. Louis, MO, USA) was added to precipitate organic matter, followed by vortexing (10 times for 2 s), and centrifugation for 7 min at 3,000 rpm. The upper green layer was carefully removed, followed by the addition of 1.5 mL of 0.2 N HCl and vortexing again. The mixture was then recentrifuged for another 7 min at the same speed. The pink upper layer was collected and transferred to 96-well microtiter plates, and the absorbance was measured at 520 nm. The amount of pyocyanin produced was calculated as the reading at 520 nm multiplied by 17.072 and represented as the resulting value per 5 mL. The results were reported as the percentage of pyocyanin production (Gupte et al., 2021).

#### 2.7.2 Pyoverdine production assay

King’s B broth was prepared following the method of King, Ward, and Raney (King et al., 1954), and the test samples were processed as previously described. Bacterial cultures were grown in King’s B broth at 37 °C with shaking at 150 rpm, and samples were collected at two time points: 12 h and 30 h. After incubation, the cell densities were measured at 600 nm using a BioTek Synergy HT microplate reader. After 12 h, 1 mL of each culture (drug-treated or untreated) was centrifuged at 10,100 × g for 10 min using an Eppendorf 5418 centrifuge (Hamburg, Germany). The resulting supernatants were transferred to 96-well microplates and their absorbance was measured at 405 nm. Pyoverdine production was calculated as 12 h λ405nm/λ600nm and 30 h λ405nm/λ600nm to get the result. Results were presented as the percentage of pyoverdine production (Sass et al., 2018).

### 2.8 Motility assays

Motility assays were performed with slight modifications based on the methods described by Niazy, Lambarte, and Alghamdi, Cai et al., and Rashid, Rao, and Kornberg (Rashid et al., 2000; Cai et al., 2020; Niazy et al., 2022). One milliliter of the bacterial suspension was transferred to ten 1.5 mL Eppendorf tubes and centrifuged at 10,100 × g for 2 min using an Eppendorf 5418 centrifuge (Hamburg, Germany). The cell pellets were then resuspended in 50 µL of PsMM containing Bu concentrations, vortexed, and 5 µL of each suspension was spotted onto motility agar plates. For swarming motility, semi-solid LB agar plates containing 0.5% agar and 5% glucose were prepared, and 5 µL of bacterial suspension was spotted on the surface of the agar, followed by incubation at 37°C for 24 h.

Swimming motility was assessed using plates composed of 1% tryptone, 0.5% NaCl, and 0.3% agarose; 5 µL of the bacterial suspension was inoculated halfway through the agar at a 45-degree angle and incubated at 30°C for 20 h. Twitching motility was evaluated on LB agar plates (1.5% agar, 3 mm thickness), where 5 µL of the suspension was applied to the bottom of the agar. After 24 h of incubation, the agar was gently removed, and the plates were stained with 2% CV for 30 min, followed by washing with distilled water. After staining and washing, motility diameters were measured, and the plates were photographed using a Nikon D3100 DSLR camera.

### 2.9 Chrome azurol S assay

Siderophore production was assessed using the SideroTec-Total™ Assay Kit (Accuplex Diagnostics Ltd., Co. Kildare, Ireland), following the manufacturer’s protocol. In summary, 96-well microtiter plates were prepared with PsMM medium, bacterial suspensions, and Bu concentrations, reaching a total volume of 200 µL per well, including appropriate controls. Plates were then incubated at 37 °C for 24 h. Following incubation, 200 µL from each well was transferred into 0.2 mL microcentrifuge tubes and centrifuged at 10,100 ×g for 5 min using a MYSPIN 12 centrifuge (Thermo Scientific). From each sample, 100 µL of the supernatant was transferred into a new 96-well plate, and assay standards were added. Absorbance was measured at 630 nm using a BioTek Synergy HT microplate reader. Then, 100 µL of the pre-mixed working reagent was added to each well, incubated for 10 min at room temperature, and absorbance was again recorded at 630 nm. Siderophore levels were evaluated both qualitatively (by visual observation) and quantitatively (by spectrophotometric readings) (Downes et al., 2023).

### 2.10 Checkerboard assays

A checkerboard microdilution assay was conducted to assess the synergistic interactions between Bu and antibiotics (gentamicin and ciprofloxacin) against the WT *P. aeruginosa* PAO1 strain. Each antibiotic was tested separately in combination with Bu. Two-fold serial dilutions were prepared for Bu (ranging from 0.125 to 128 µg/mL) and for the antibiotics (ranging from 0.125 to 8 µg/mL). The assay was performed in 96-well microtiter plates containing PsMM, Bu dilutions along the x-axis, antibiotic dilutions along the y-axis, and bacterial suspension to reach a final volume of 200 µL per well. The first well included untreated bacterial cells as a negative control, while the first row and first column represented Bu alone and the antibiotics alone, respectively, serving as positive controls. The plates were incubated at 37 °C for 18–20 h. Bacterial growth was measured by recording absorbance at 600 nm using a BioTek Synergy HT microplate reader. To correct for baseline variation, OD values at 0 h were subtracted from post-incubation readings for all wells (Bellio et al., 2021). Data analysis was performed using SynergyFinder (https://synergyfinder.fimm.fi) (Ianevski et al., 2022). The minimum inhibitory concentration (MIC) was defined as the lowest drug concentration producing 80% growth inhibition. The fractional inhibitory concentration (FIC) for Bu was calculated as the MIC of Bu in combination divided by the MIC of Bu alone. Similarly, FIC values for gentamicin and ciprofloxacin were determined. The fractional inhibitory concentration index (ΣFICI) was obtained by summing the FICs of the two agents. ΣFICI values were interpreted as follows: ≤0.5 indicating synergy, >0.5 to ≤4 indicating indifference, and >4 indicating antagonism (Foweraker et al., 2009).

### 2.11 *In silico* molecular docking prediction of Bu in PAO1 proteins

A list of commonly known genes associated with pigment production and biofilm formation was selected, and docking affinities and interactions were further correlated with the potential effects of Bu on PAO1 pathogenicity (Ding et al., 2018; Díaz-Pérez et al., 2023). The selected proteins (lasR, pqsE, qscR, and alg44) were chosen as docking targets based on published pathway and regulatory studies; they are known regulators of these pathways and are commonly explored in experimental and computational studies screening anti-virulence agents for potential activity against PAO1 (Bottomley et al., 2007; Zender et al., 2016; Ding et al., 2018; Poulin et al., 2021; Taylor et al., 2022; Manu et al., 2025). The crystal structures of alginate synthase regulation (alg44, Protein Data Bank (PDB) ID: 4RT0), quorum-sensing transcriptional regulator (lasR, PDB ID: 2UV0), PQS response protein (pqsE, PDB ID: 5HIQ), and quorum-sensing regulator (qscR, PDB ID: 6CC0) were obtained from the Protein Data Bank (RCSB PDB, 2025; Berman et al., 2000). All protein structures were prepared using the AutoDock tool protocol and the Protein Repair and Analysis Server (PRAS) (Forli et al., 2016; Nnyigide et al., 2022). The co-crystallized ligand-binding sites of alg44, lasR, pqsE, and qscR were determined using Discovery Studio Visualizer (Dassault Systèmes BIOVIA, Discovery Studio 2024 Client version 24.1.0) (Visualizer, 2005). The 3D structure of Bu was downloaded from the PubChem database (PubChem ID: 2478) (Kim et al., 2016). The ligand was optimized using Avogadro software (version 1.2.0) (Avogadro Team, 2025). and exported to mol2 format. The protein and ligand PDBQT files for molecular docking were generated using the AutoDock tool. The receptor parameters for Vina molecular docking were outlined in **Supplementary Table S1**. Molecular docking was performed using AutoDock Vina software (version 1.2.7) (Trott and Olson, 2010). The best docking scores were selected, and the output complex poses were computed using PyMol software (version 3.0.3). Discovery Studio Visualizer software (version 24.1.0) was used to generate hydrogen-bond surface interactions and perform protein–ligand interaction analysis, and ChimeraX software (version 1.7.1) was used to generate 3D conformations.

### 2.12 Statistical analysis

All experiments were independently repeated with three biological replicates in triplicate, and the results were reported as mean values with standard deviation (± SD). Statistical analyses and graphs were performed using GraphPad Prism for Windows (version 10.5.0 (774)) (Boston, MA, USA; www.graphpad.com). In certain experiments, one-way analysis of variance (ANOVA) followed by Dunnett’s *post hoc* test was applied to determine statistically significant differences between treated and untreated groups for both bacterial strains. A p-value of ≤0.05 was considered statistically significant.

## 3 Results

### 3.1 MIC, MBC, and growth kinetics

**Figure 2:**
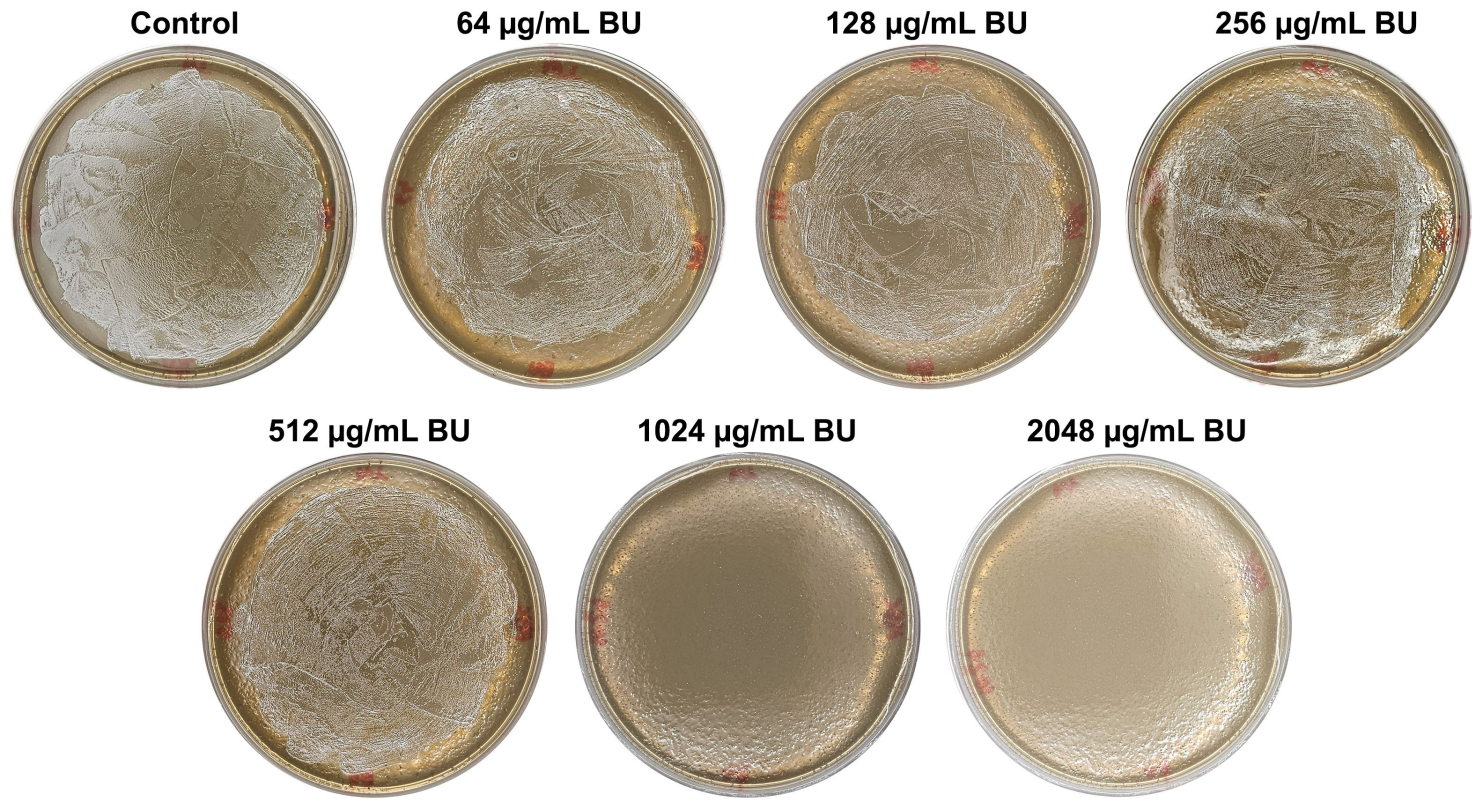
MBC determination of Bu on *P. aeruginosa* PAO1.

The percentage inhibition of *P. aeruginosa* PAO1 treated with Bu was calculated at each concentration (0.5, 3, 18, and 108 µg/mL) to determine the MIC. PAO1 treated with 108 µg/mL Bu showed 84.45% inhibition. Significant inhibition was observed when treating PAO1 with 18 µg/mL and 108 µg/mL Bu (*p* < 0.0001). Overall, the percentages of PAO1 growth inhibition are presented in **Supplementary Table S2**. However, the MBC for PAO1 treated with Bu was >512 and <1,024 µg/mL (**Figure 2**).

The growth curve of WT PAO1 (**Supplementary Figure 1**) showed a slower growth rate, especially when exposed to the highest concentration of Bu (108 µg/mL) compared withwith the untreated control and lower concentrations. The growth curves also showed that bacterial growth gradually decreased in the presence of Bu compared with the control.

### 3.2 Biofilm quantification and viability

#### 3.2.1 Biofilm formation

**Figure 3:**
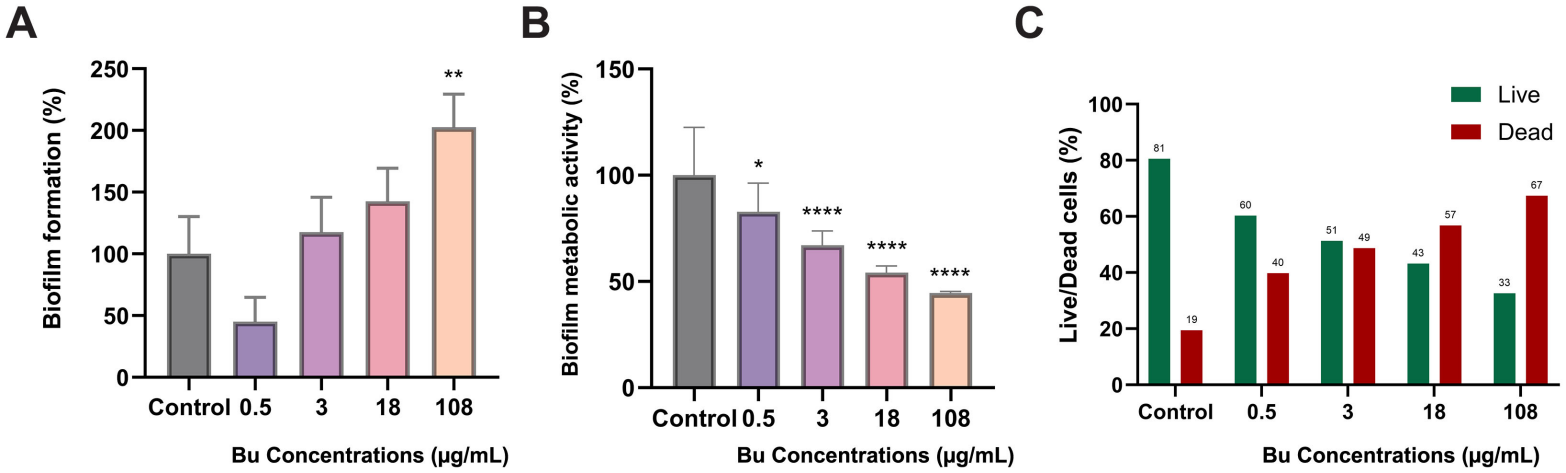
Effects of Bu treatment at different concentrations on the biofilm of *P. aeruginosa* PAO1. **(A)** Effects of Bu concentrations on biofilm formation of *P. aeruginosa* PAO1 after 48 h Data are expressed as the mean ± SD (n = 9); **p < 0.01. **(B)** Effects of the different concentrations of Bu on the metabolic activity of PAO1 biofilm using resazurin assay. Data are expressed as the mean ± SD (n = 9). *p < 0.05; ****p < 0.0001. **(C)** Cell viability of PAO1 biofilm in percentage representing live cells (green bars) and dead cells (red bars). Data are expressed as the mean (n = 12).

PAO1 treated with 0.5 µg/mL Bu showed aa reduction in biofilm formation of about 55% (**Figure 3A**). However, increasing Bu concentrations enhanced biofilm formation, with108 µg/mL Bu treatment showing a significant increase in biofilm formation, approximately doubling it (102.5% increase).).

#### 3.2.2 Resazurin assay

A significant, dose-dependent reduction was observed in the metabolic activity of the PAO1 biofilm after treatment with different Bu concentrations compared with the untreated samples, as measured by the resazurin reduction assay (**Figure 3B**). The viable metabolic activity was reduced by about 55% at the highest concentration (*p* < 0.0001). A slight but statistically significant reduction was detected at 0.5 µg/mL (82.8 ± 13.5%, *p* < 0.05), followed by a substantial drop at 3 µg/mL (67.1 ± 6.7%, *p* < 0.0001). At higher concentrations, Bu exerted a marked inhibitory effect, reducing metabolic activity to 54.2 ± 3.1% (*p* < 0.0001) and 44.6 ± 0.07% (*p* < 0.0001) at 18 µg/mL and 108 µg/mL, respectively.

#### 3.2.3 Biofilm viability and structure

A significant reduction in live cells of PAO1 was observed across all samples treated with Bu. The percentage of dead cells in the control sample was 19%, which increased to 40%, 49%, 57%, and 67% at concentrations of 0.5, 3, 18, and 108 µg/mL Bu, respectively. Correspondingly, the percentage of live cells decreased from 81% in the untreated sample to 60%, 51%, 43%, and 33% at the same Bu concentrations (**Figure 3C**).

**Figure 4:**
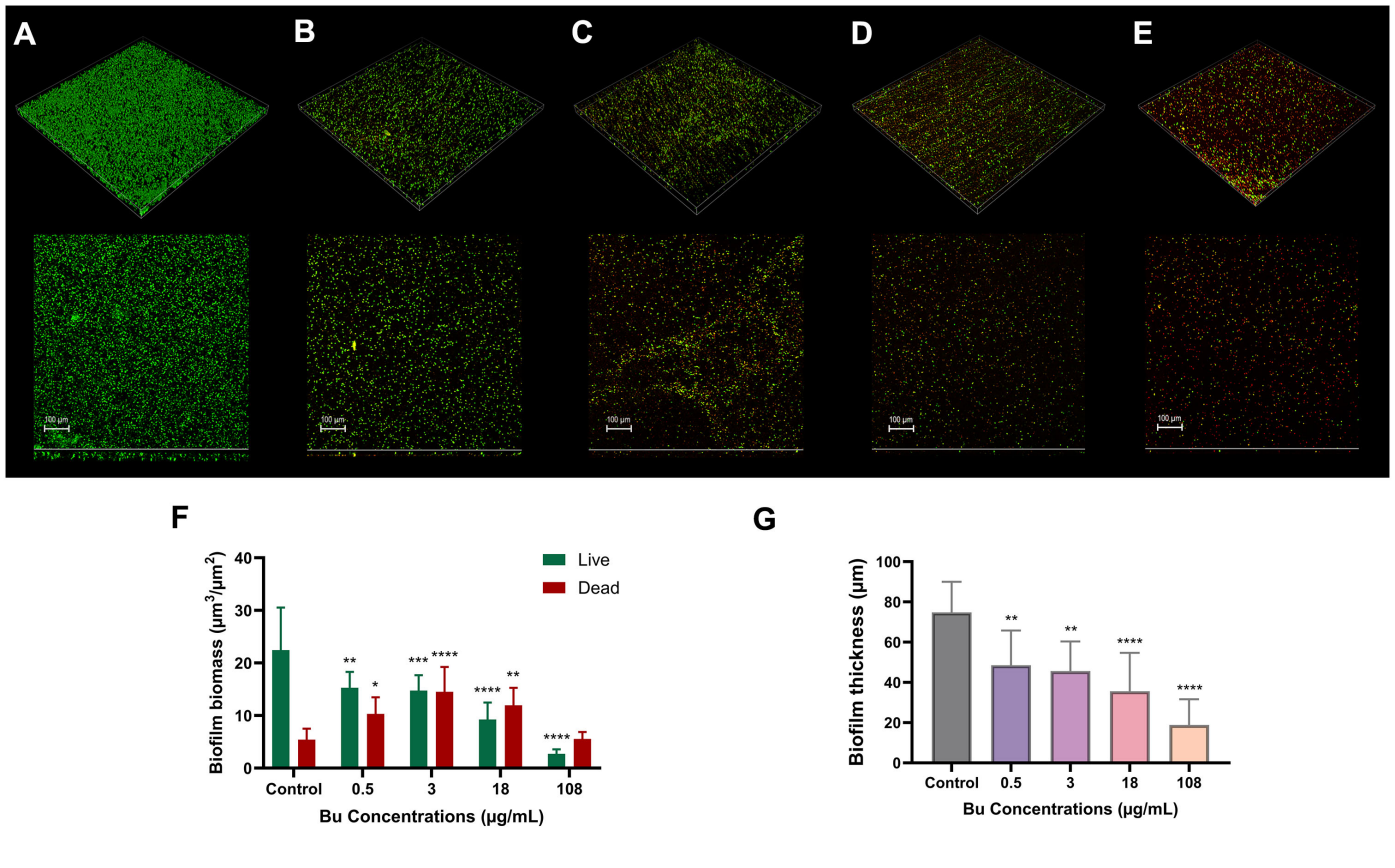
Representative CLSM images of PAO1 biofilms after 48 h of Bu treatment (100 µm scale). Biofilms of **(A)** untreated control and treatment with **(B)** 0.5, **(C)** 3, **(D)** 18, and **(E)** 108 µg/mL Bu. **(F)** Biofilm biomass measurements expressed in µm³/µm². **(G)** Biofilm thickness expressed in µm. Data are expressed as the mean ± SD (n = 12). *p < 0.05; **p < 0.01; ***p < 0.001; ****p < 0.0001.

The biomass of live cells significantly decreased in a dose-dependent manner (**Figures 4A–E**), from 22.44 ± 8 µm^3^/µm^2^ for the untreated sample to 2.7 ± 0.9 µm^3^/µm^2^ at the highest Bu concentration (**Figure 4F**). CLSM measurements revealed a significant concentration-dependent reduction in biofilm thickness as Bu concentrations increased (**Figures 4A–E**). Thickness for the untreated sample was 75 ± 15.26 µm, whereas treated samples showed progressive decreases to 49 ± 17.30 µm, 46 ± 14.74 µm, 36 ± 19.09 µm, and 19 ± 12.78 µm at 0.5, 3, 18, and 108 µg/mL Bu, respectively (**Figure 4G**).

### 3.3 Hemolytic activity

Hemolytic activity was assessed to determine whether Bu influences the cytolytic phenotype of PAO1. The results showed clear β-hemolysis in both Bu-treated and untreated samples, indicating that Bu did not alter hemolytic activity under the tested conditions. These findings suggest that, unlike other virulence-associated traits evaluated in this study, hemolysis appears to be unaffected by Bu exposure (**Supplementary Figure 2**).

### 3.4 Pigments production

#### 3.4.1 Pyocyanin production

**Figure 5:**
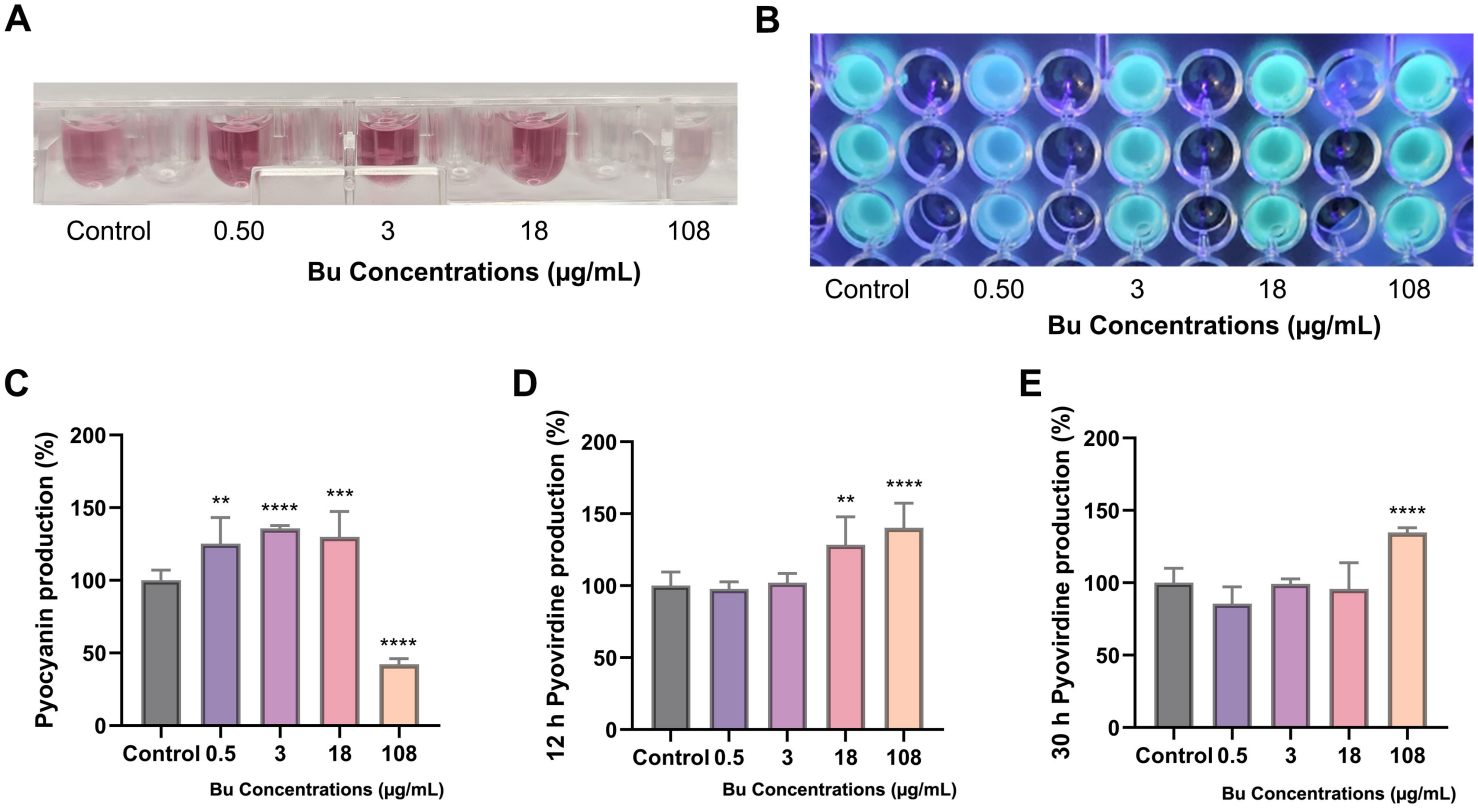
Effects of Bu exposure on pigment production in *P. aeruginosa* PAO1. **(A)** Representative image of pyocyanin produced after Bu treatment in liquid King’s A medium. **(B)** Representative image of fluorescence detection of PAO1 after Bu treatment in King’s B medium under UV light (30 h). **(C)** Effects of Bu concentrations on pyocyanin production compared withwith control. Data are expressed as the mean ± SD (n = 9); **p < 0.01; ***p < 0.001; ****p < 0.0001. **(D)** Percentage of pyoverdine produced after 12 h of Bu exposure. **(E)** Percentage of pyoverdine produced after 30 h of Bu exposure. Data are expressed as the mean ± SD (n = 9); **p < 0.01; ****p < 0.0001.

The results demonstrated that 108 µg/mL Bu exposure significantly reduced the PAO1 virulence factor pyocyanin production by 57% compared with the control (**Figure 5C**). In contrast, lower concentrations of Bu promoted pyocyanin production in PAO1 relative to the control. Exposure to different concentrations of Bu had a statistically significant effect on PAO1 pyocyanin pigmentation (**Figure 5A**).

#### 3.4.2 Pyoverdine production

Results of pyoverdine production by *P. aeruginosa* PAO1 treated with Bu were assessed at two time points: 12 and 30 h (**Figures 5D, E**). PAO1 treated with 108 µg/mL Bu showed a significant increase in pyoverdine production of about 40% after 12 h of incubation and about 35% after 30 h of incubation compared with the control. After 12 h of exposure, a significant increase in pyoverdine production was observed at higher Bu concentrations, reaching 128.34 ± 19.5% (*p* < 0.01) and 140.2 ± 17.17% (*p* < 0.0001) at 18 µg/mL and 108 µg/mL, respectively. Similarly, after 30 h, a marked elevation was detected at 108 µg/mL Bu (134.8 ± 3.3%, *p* < 0.0001) compared with the untreated control (**Figure 5B**).

### 3.5 Motility

**Figure 6:**
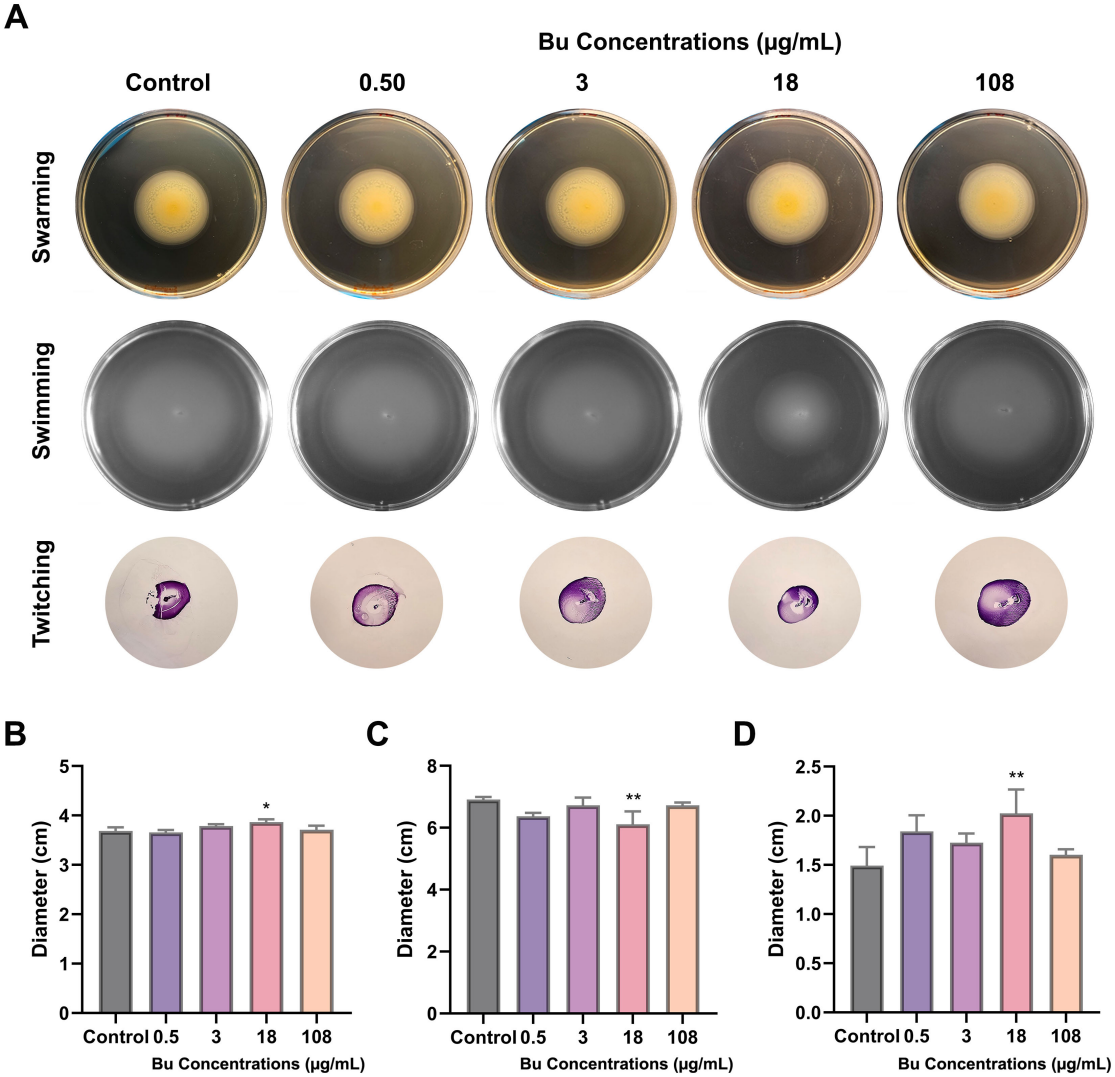
Effects of Bu treatment at different concentrations on the motility of *P. aeruginosa* PAO1. **(A)** Representative images of PAO1 swarming, swimming, and sub-surface twitching after Bu treatment. **(B)** Mean swarming motility of PAO1 treated with Bu. Data are expressed as the mean ± SD; *p < 0.05. **(C)** Mean swimming motility of PAO1 treated with Bu. Data are expressed as the mean ± SD; **p < 0.01. **(D)** Mean twitching motility of PAO1 treated with Bu. Data are expressed as the mean ± SD; **p < 0.01.

There was a significant increase in swarming for PAO1 treated with 18 µg/mL Bu of approximately 4.9% (*p* < 0.05) compared with the control (**Figure 6B**). Moreover, swimming motility of PAO1 treated with 18 µg/mL Bu was significantly reduced by 11.58% (*p* < 0.01) (**Figure 6C**), and sub-surface twitching motility was significantly increased by 35.51% (*p* < 0.01) (**Figure 6D**) compared with the respective controls. Other concentrations did not produce statistically significant changes in swarming, swimming, or twitching motilities (**Figure 6A**).

### 3.6 Siderophore production

**Figure 7:**
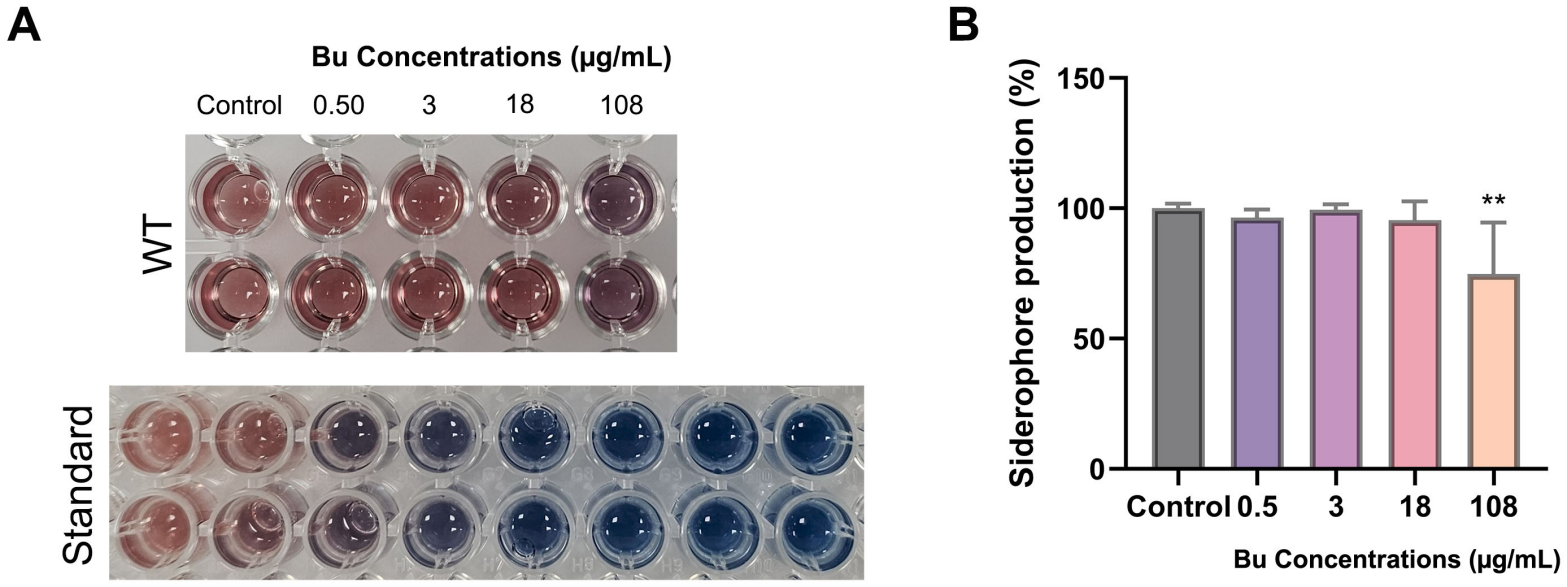
The ability of PAO1 treated with Bu concentrations of chelating Fe³^+^ from the chromophore. **(A)** Representative images of PAO1 after the Bu treatment. **(B)** The percentage of siderophores produced by PAO1 after Bu treatment. Data are expressed as the mean ± SD (n = 9); **p < 0.01.

PAO1 treated with 108 µg/mL Bu was capable of chelating Fe³^+^ from the chromophore, resulting in a significant reduction of 25% compared with the untreated sample (p < 0.01) (**Figure 7B**). This inhibitory effect was concentration-specific, as lower Bu concentrations produced no significant changes in siderophore levels. Overall, Bu affected siderophore production only at the highest concentration tested, indicating a limited but measurable impact on PAO1’s iron-acquisition capacity (**Figure 7A**).

### 3.7 Checkerboard assay

**Figure 8:**
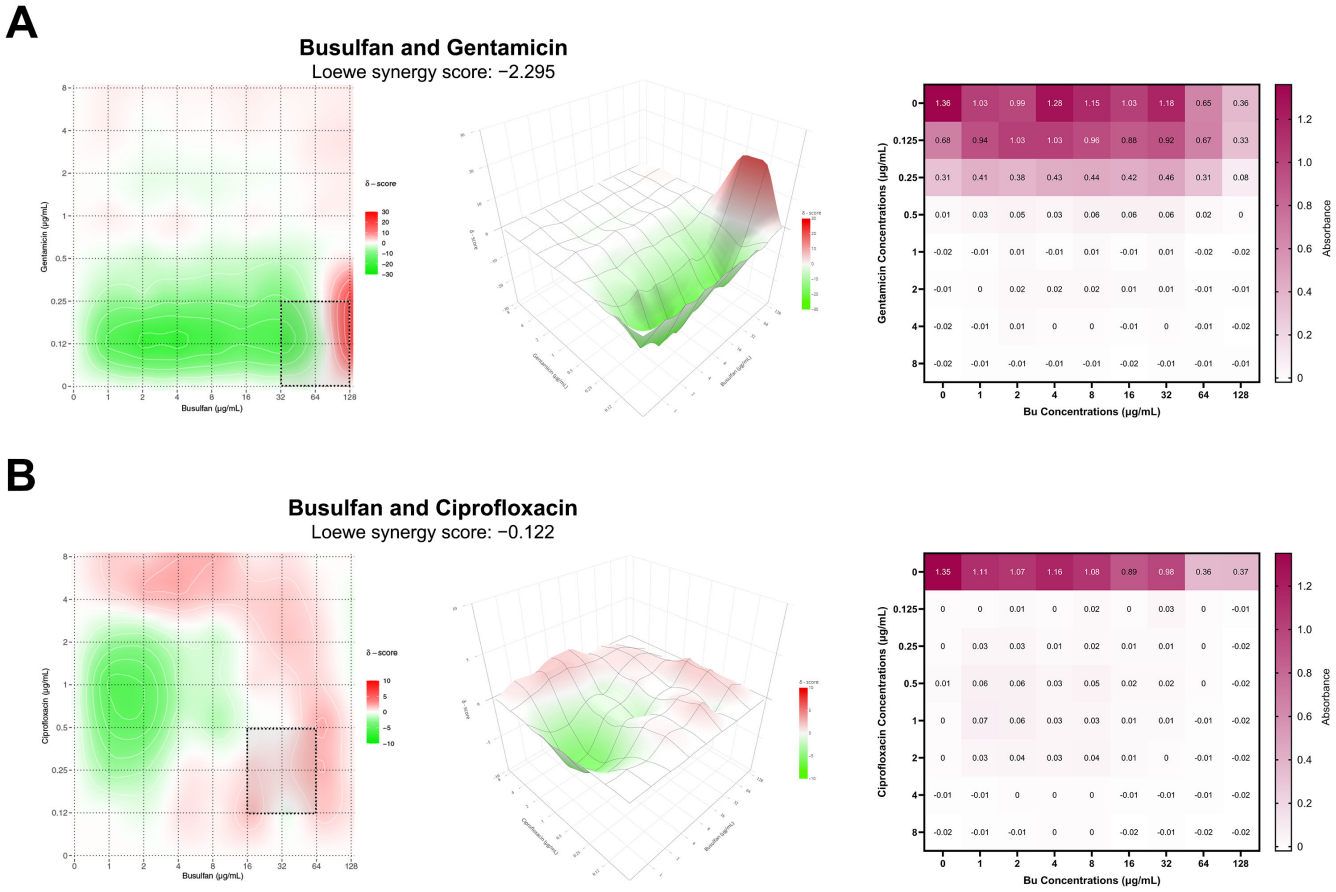
**(A)** Synergy analysis of gentamicin–busulfan combination. **(B)** Synergy analysis of busulfan–ciprofloxacin combination. The left panel shows the 2D synergy maps (dotted box indicates the most synergistic region). The middle panel shows the 3D synergy maps. The right panel shows a heatmap of OD_600_ values from the checkerboard assay in a 96-well microtiter plate. Busulfan concentrations (0–128 µg/mL) are arranged along the horizontal axis, and antibiotic concentrations (0–8 µg/mL) along the vertical axis. Darker shades indicate higher absorbance values, reflecting greater bacterial growth.

Bu was tested in combination with gentamicin to evaluate potential synergy against PAO1. The combination exhibited a Loewe synergy score of −2.295 and resulted in a fractional inhibitory concentration index (FICI) of 1, indicating an indifferent interaction overall. However, gentamicin showed moderate synergy at concentrations beyond 128 µg/mL Bu, which might further strengthen this interaction pattern at higher doses (**Figure 8A**). Moreover, ciprofloxacin showed an indifferent effect when combined with Bu, giving a Loewe synergy score of −0.122 with an FICI of 1 (**Figure 8B**). Overall, the combination data indicate that Bu does not significantly modulate antibiotic activity at sub-inhibitory concentrations, although changes observed at higher doses suggest that further exploration of potential stress-related interactions is necessary.

### 3.8 Molecular docking study

**Figure 9:**
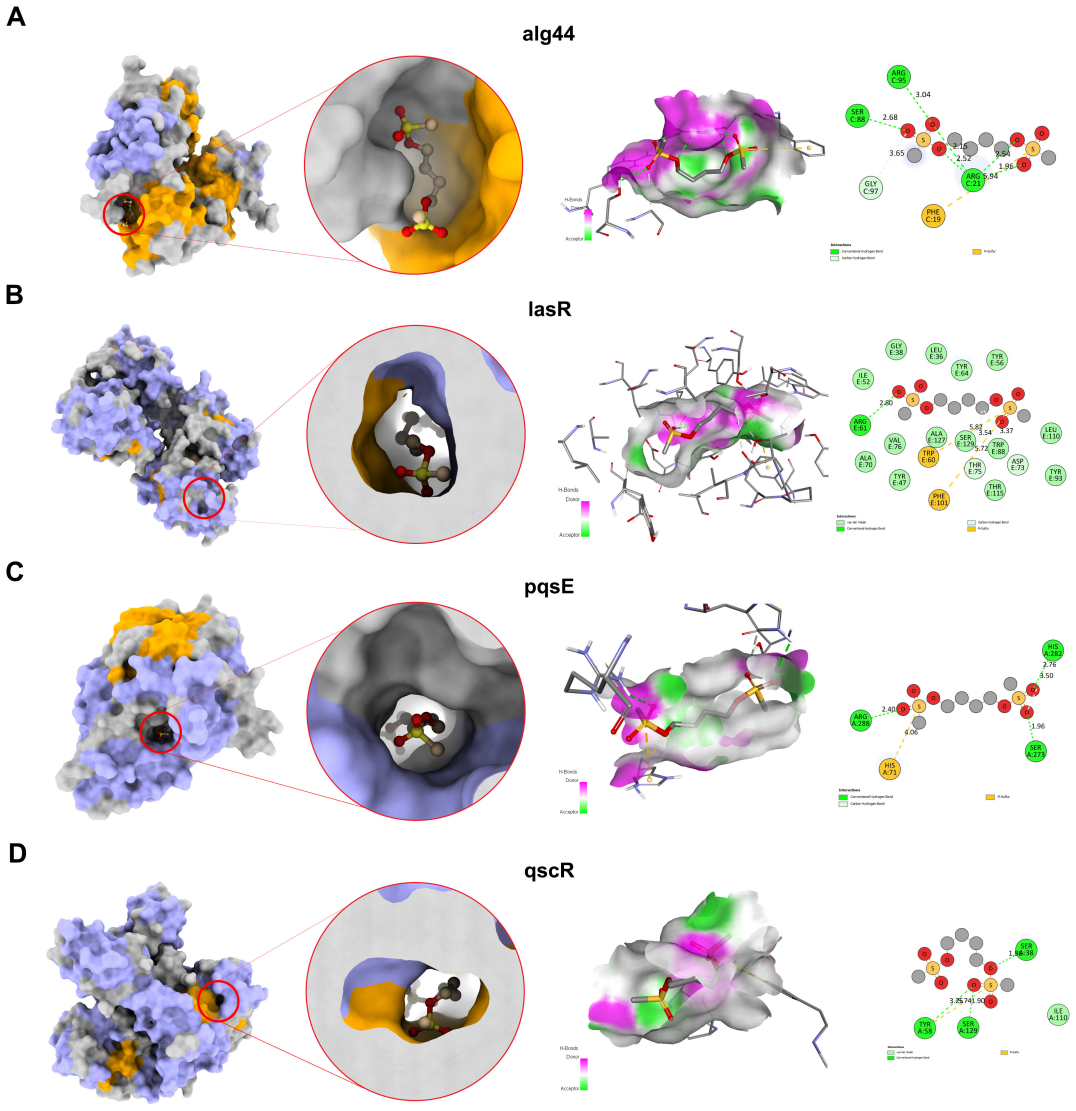
Molecular docking of Bu with PAO1 receptors. Interaction of Bu with **(A)** alg44, **(B)** lasR, **(C)** pqsE, and **(D)** scR (interaction panels). Left panel: protein surface with predicted binding site (red circle); helices (purple), β strands (orange), loops (gray). Middle panel: hydrogen-bond interactions (green dashed lines) and π–stacking interactions (yellow dashed lines). Right panel: interaction diagram of Bu and PAO1 receptors. Pink areas denote hydrogen-bond donors; green areas denote hydrogen-bond acceptors.

Molecular docking studies were performed on four selected PAO1 proteins (alg44, lasR, pqsE, and qscR) with Bu (**Figure 9**). This was done to identify the potential mechanism of Bu-mediated reduction in PAO1 virulence based on the observed effects on pyocyanin and siderophore production and on swarming and swimming motilities. The binding affinities for each receptor are shown in **Supplementary Table S3**. AutoDock Vina predicted similar docking scores across alg44, lasR, pqsE, and qscR of −5.6, −6.4, −5.7, and −5.9 kcal/mol, respectively. The docking score for lasR was the most negative, whereas that for alg44 was the least negative. Alg44 showed the highest total number of hydrogen bonds compared with lasR, pqsE, and qscR.

## 4 Discussion

This study evaluated the effectiveness of the alkylating agent Bu at various concentrations against *P. aeruginosa*, with an emphasis on its impact on antibacterial activity and virulence factor production. In this study, the MIC of Bu against PAO1 was 108 µg/mL, while the MBC was >512 and <1,024 µg/mL. To date, no published studies have reported on the antibacterial activity of Bu against *P. aeruginosa*. However, the present findings demonstrate measurable inhibitory effects under the tested conditions. Soo et al. (2017) proposed that Bu’s ability to induce DNA damage may contribute to its antibacterial effects against *S. aureus*, *E. faecium*, and *P. aeruginosa* (Soo et al., 2017).

In this study, CLSM live/dead analysis revealed a significant reduction in biofilm thickness and viable cell populations The percentage of dead cells increased from 19% in untreated samples to 67% in samples treated with 108 µg/mL Bu. Similarly, the resazurin assay showed a comparable trend to the CLSM results, with metabolic viability decreasing by approximately 55% at the highest concentration (*p* < 0.0001). The correlation between the metabolic and imaging data confirms that Bu significantly compromises biofilm viability, leading to a substantial loss of metabolically active cells within the biofilm matrix, confirming the effect of Bu on reducing biofilm viability.

However, increasing concentrations of Bu in the culture medium of PAO1 resulted in enhanced biofilm formation, as indicated by crystal violet staining after 48 h of incubation. The observed increase in biofilm formation with increasing drug concentrations may be attributed to stress-induced adaptive responses, including enhanced persister cell formation. Persister cells are temporarily dormant bacterial variants that survive treatment without being genetically resistant. They remain inactive in the presence of high drug concentrations and can later reactivate once the antibiotic pressure is removed (Jangra et al., 2022).

This apparent contradiction reflects the distinction between total biofilm material and viable cellular content. The increase in biomass may be due to the accumulation of extracellular matrix, dead cells, and debris, rather than an expansion of the living biofilm. Such changes are consistent with the maturation or early dispersal phase of biofilm development, where structural remnants persist even as cell viability declines. Latka and Drulis-Kawa demonstrated that agents degrading biofilm matrix components can cause a “false-positive” increase in biomass readings via CV, while viability and metabolic assays such as live/dead CLSM and resazurin biofilm quantification allow for more accurate interpretation of biofilm dynamics (Latka and Drulis-Kawa, 2020).

Although clinical data on Bu–antibiotic combinations are lacking, several studies show that repurposed anticancer agents can act as adjuvants to conventional antibiotics. Cheng et al. reported that biofilm-associated bacteria require much higher MICs and MBCs than planktonic cells, making complete biofilm eradication with single antibiotics difficult. Therefore, combining antibiotics or adding adjunct compounds has gained attention as a promising strategy against biofilm-related infections (Cheng et al., 2022). Niazy et al. demonstrated that 5-fluorouracil reduced the biomass of *P. aeruginosa* biofilms by approximately 70% at sub-inhibitory concentrations (Niazy et al., 2024a). Additionally, 5-fluorouracil in combination with gentamicin resulted in synergistic biofilm inhibition. Gnanadhas et al. also showed that L-methionine disrupted established *P. aeruginosa* biofilms and enhanced ciprofloxacin-mediated biofilm clearance and survival in a mouse infection model (Gnanadhas et al., 2015). Torres et al. found that auranofin–colistin and clomiphene citrate–colistin combinations have significant antibiofilm activity against preformed *P. aeruginosa* biofilms (Torres et al., 2018). Moreover, mitomycin C, a DNA-alkylating anticancer agent similar to Bu, also requires high MICs, but Schweizer et al. reported that combining mitomycin C with a tobramycin–ciprofloxacin hybrid (TOB-CIP) reduced the MICs against MDR *P. aeruginosa* by up to 128-fold (Schweizer, 2019). Overall, these findings support combination therapy as a more effective strategy than monotherapy for enhancing the eradication of both biofilm-associated and planktonic bacterial cells. Further *in*-depth *in vivo* validation and safety assessments are warranted to determine whether such combinations can be translated into clinically applicable antibiofilm interventions.

Moreover, growth curve analysis of PAO1 confirmed a reduction in growth rate in a dose-dependent manner approximately 7 h after exposure to higher concentrations of Bu. However, the organism demonstrated the capacity to recover its growth, which may be attributed to Bu’s reported stability of 8 h at 20 ± 55°C according to Pierre Fabre Laboratories, as mentioned by Guichard (Guichard et al., 2017). Despite Bu’s classification as a hydrophobic compound, it is possible that the compound may traverse the outer membrane bilayer of *P. aeruginosa* via a self-enhancing uptake mechanism, potentially contributing to its observed antibacterial properties (Nikaido, 2003). Moreover, the stability of Bu may have contributed to the observed results. Exposure to 0.5 µg/mL Bu demonstrated a consistent inhibitory effect compared with other concentrations. This effect was observed across nearly all experiments in this study and may be linked to strain-specific stress responses and adaptive mechanisms in gram-negative bacteria (Chen et al., 2020). Additionally, variations in experimental conditions, such as the type of growth medium used, likely influenced the observed outcomes (Lozano et al., 2020).

An increase in hemolysis was observed at low concentrations of Bu. This response is likely due to a stress-induced activation of quorum-sensing systems at sub-inhibitory levels, promoting virulence factor production (Eltayb et al., 2022). Although the results observed at 108 µg/mL appeared β-hemolytic, it is possible that the drug lost its activity over time, allowing the organism to recover and resume growth.

The results of pigment analysis revealed that pyocyanin production by PAO1 increased in a dose-dependent manner at low Bu concentrations, consistent with a quorum-sensing–mediated stress response. At sub-inhibitory levels, *P. aeruginosa* may activate virulence mechanisms, including pyocyanin biosynthesis, to enhance its survival (Eltayb et al., 2022). In contrast, the observed decrease at 108 µg/mL Bu likely reflects a transition from stress-induced virulence activation to metabolic suppression as the drug approaches inhibitory levels. Furthermore, the observed dose-dependent increase in pyoverdine production by PAO1 likely reflects a stress adaptation mechanism. Under sub-inhibitory concentrations of Bu, bacteria may experience functional iron limitation or redox imbalance, leading to upregulation of siderophore synthesis (Kang et al., 2018).

The fluctuation in motility observed across different Bu concentrations may reflect a dynamic regulatory balance between motility and stress-adaptation pathways in *P. aeruginosa*. This includes quorum-sensing variability and lifestyle switching between planktonic and biofilm states in response to drug stress, as mentioned previously. Environmental or technical sensitivity of motility assays may also contribute to the observed pattern (Moradali et al., 2017).

The results demonstrated that PAO1 treated with higher Bu concentrations was capable of chelating Fe³^+^ from the chromophore. The observed decrease in CAS assay activity, alongside an increase in pyoverdine production, suggests a functional decoupling between siderophore quantity and iron-chelating capacity. The pyoverdine measurement quantifies the total amount of fluorescent pyoverdine produced, regardless of its iron-loading status, whereas the CAS assay specifically reflects iron-chelating capacity. Therefore, bacteria under drug-induced stress might produce altered pyoverdine variants with reduced iron affinity, which are still fluorescent but less effective in iron scavenging, thus reducing CAS activity (Dell’Anno et al., 2022). Moreover, pyoverdine production was observed in King’s B medium, while siderophore activity was measured by CAS assay in PsMM. This may be attributed to the distinct composition and iron availability in each medium. While King’s B supports robust growth and enhances fluorescent pyoverdine synthesis, PsMM’s more restrictive environment, combined with Bu-induced stress, likely led to the accumulation of iron-saturated (holo-) pyoverdine, which does not contribute to CAS-based iron chelation (Gomes et al., 2024). These findings highlight the importance of medium selection and the need to assess both siderophore production and functionality when interpreting results, as demonstrated by the use of King’s B medium for pyoverdine quantification and PsMM for CAS assay analysis.

It is proposed that the antibacterial effect of Bu may be enhanced when combined with clinically approved antibiotics that act on different bacterial targets. For instance, gentamicin disrupts protein synthesis, while ciprofloxacin inhibits DNA gyrase, thereby interfering with bacterial DNA replication (Holger et al., 2022; Kunz Coyne et al., 2024). The strategy of combining antibiotics with additional agents has shown effectiveness in both *in vitro* and *in vivo* studies. Svedholm et al. showed that combining mitomycin C with gentamicin (mitomycin C–antibiotic therapy) resulted in synergistic inhibition of *P. aeruginosa in vitro. In vivo*, the combination therapy demonstrated increased effectiveness against *Galleria mellonella* larvae infected with *P. aeruginosa* compared with monotherapy (Svedholm et al., 2024). Other studies reported that using phage–antibiotic therapy against *P. aeruginosa* significantly improved bacterial eradication (Holger et al., 2022; Kunz Coyne et al., 2024). In this study, gentamicin exhibited a moderate synergistic effect at higher concentrations of Bu, suggesting that extending the Bu concentration beyond 128 µg/mL may potentially enhance the synergistic interaction. Gentamicin exerts its antibacterial effect by binding to the 30S subunit of the bacterial ribosome, causing misreading of mRNA and resulting in the production of faulty proteins (Blair et al., 2015). This approach makes it more difficult for bacteria to develop resistance to both agents simultaneously, which may enhance the overall therapeutic efficacy of the combination, particularly if higher concentrations of Bu are explored.

Furthermore, the molecular docking study that was carried out using key regulatory proteins of motility and virulence factors (alg44, lasR, pqsE, and qscR) (Bottomley et al., 2007; Whitney et al., 2015; Zender et al., 2016; Wysoczynski-Horita et al., 2018) aimed to understand the phenotypic effects of Bu against PAO1 as observed in different assays. The docking analysis demonstrated a moderate binding affinity to alg44 (−5.6 kcal/mol), suggesting interference with alginate regulation and surface-associated motility via cyclic di-GMP signaling [Jangra et al., 2022]; and to pqsE (−5.7 kcal/mol), which is linked to controlled-virulence quorum-sensing pathways. The moderate–strong interaction with LasR (−6.4 kcal/mol), a quorum-sensing regulator controlling several virulence factors, may contribute to the observed phenotypic reductions influencing motility and siderophore production (Jeong et al., 2023; Manu et al., 2025). Moreover, the interaction with qscR (−5.9 kcal/mol), a QS anti-activator, suggests potential modulation of QS timing that could disrupt virulence gene activation (Ding et al., 2018). These results suggest that Bu’s mechanistic effects on multiple PAO1 targets may reduce its pathogenicity.

Although Bu exhibits limited dilution stability in saline, with rapid degradation, a recent study conducted by Xu et al. (2025) demonstrated that embedding Bu into a sulfobutylether-*β*-cyclodextrin (Bu–SBE-*β*-CD) cavity structure using inclusion complex technology and freeze-drying improved Bu stability without compromising its physicochemical or therapeutic properties (Xu et al., 2025). Moreover, nanotechnology and prodrugs are two approaches that have also been explored by researchers. For example, utilizing Bu prodrugs formulated within nanocarriers such as hydrogels or nanoparticle systems could potentially improve stability and enable site-specific transport (Ioele et al., 2022). However, additional experiments, such as cytotoxicity assays on non-tumor human cells and gene-expression studies, are needed to better assess its safety and antimicrobial effects.

Our data highlight that Bu can modulate key virulence factors of *P. aeruginosa*, indicating that aspects of its mechanism such as its alkylating activity or its effects on DNA- and metabolism-associated pathways may represent novel antimicrobial targets. Similar repurposing frameworks have been applied successfully to other anticancer agents with documented antimicrobial or antivirulence properties, supporting the conceptual validity of this approach. These findings, while based on a laboratory model, contribute to the growing interest in exploring its antibacterial potential. While the MIC of Bu surpasses its usual therapeutic plasma levels (0.5–1.5 µg/mL), these findings indicate a potential role for Bu in affecting *P. aeruginosa*, though further investigations across different models and clinical settings are essential to establish its *in vivo* applicability and safety profile (Svedholm et al., 2024).

This study has some limitations. The findings are based on a laboratory model, which may not fully represent *in vivo* conditions. This limitation restricts the direct extrapolation of the data to clinical scenarios, where factors such as host immune response, pharmacokinetics, and tissue distribution play critical roles. Additionally, further investigations are necessary to explore localized delivery methods or structural modifications of Bu to enhance its antimicrobial potency while minimizing systemic exposure, such as nanocarriers mentioned previously that enable site-specific transport and enhance busulfan stability, as well other innovative approaches. More comprehensive studies including gene-expression analyses and evaluations across different models and clinical isolates are essential to establish its efficacy, safety profile, and potential role in affecting *P. aeruginosa*. Moreover, evaluating busulfan’s interaction with other antimicrobial agents could reveal synergistic effects that may lower the required dosage and improve therapeutic outcomes. Overall, while the current findings contribute valuable preliminary insights into busulfan’s potential as an antimicrobial agent, these limitations underscore the need for more comprehensive and translational studies.

## 5 Conclusion

Busulfan shows potential as a therapeutic candidate against *P. aeruginosa* by inhibiting biofilm formation, decreasing viable cell counts, and reducing the production of key virulence factors such as pyocyanin and siderophores. The overall findings are promising and warrant further investigation into the effectiveness of Bu as an antimicrobial agent against *P. aeruginosa*, which could potentially support its future application as a therapeutic agent.

## Data Availability

The raw data supporting the conclusions of this article will be made available by the authors, without undue reservation.
