# Supplementary material for: Busulfan inhibits Pseudomonas aeruginosa growth and reduces biofilm biomass and pyocyanin production
Source: Front Cell Infect Microbiol. 2026 Jan 2;15:1721773. doi: 10.3389/fcimb.2025.1721773 (PMC12808452; doi:10.3389/fcimb.2025.1721773)
Supplement: Supplementary file 1 [file DataSheet1.docx]

**Supplementary Materials**

**Supplementary Figure 1.** Effects of Bu treatment on the growth of *P. aeruginosa* PAO1.

**
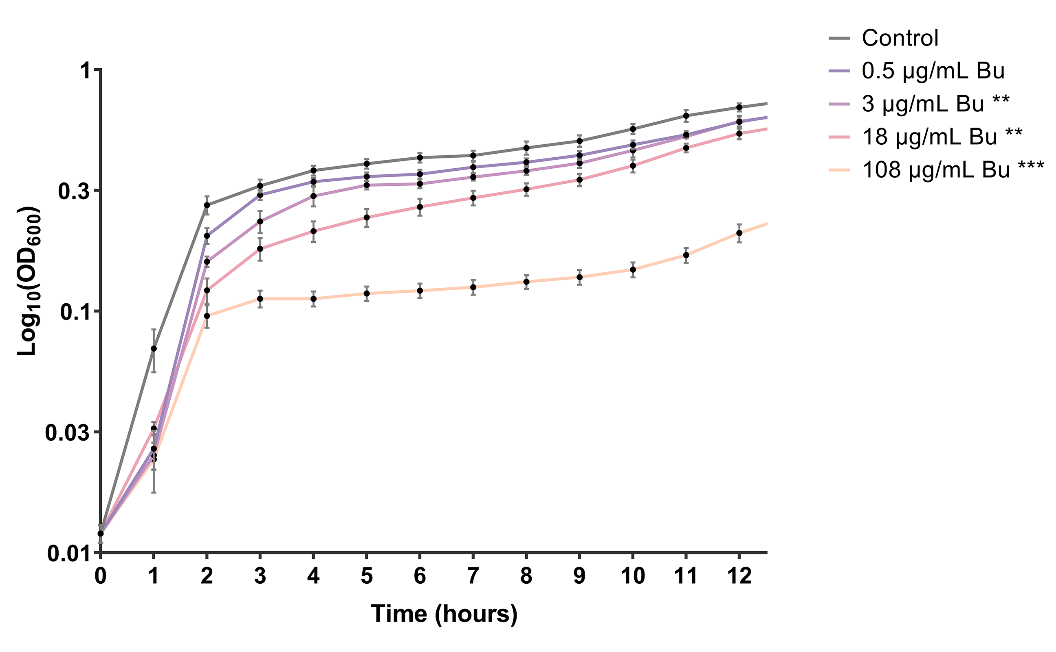
**

**Supplementary Figure 2.** Hemolysis of PAO1 treated with different concentrations of Bu.

**
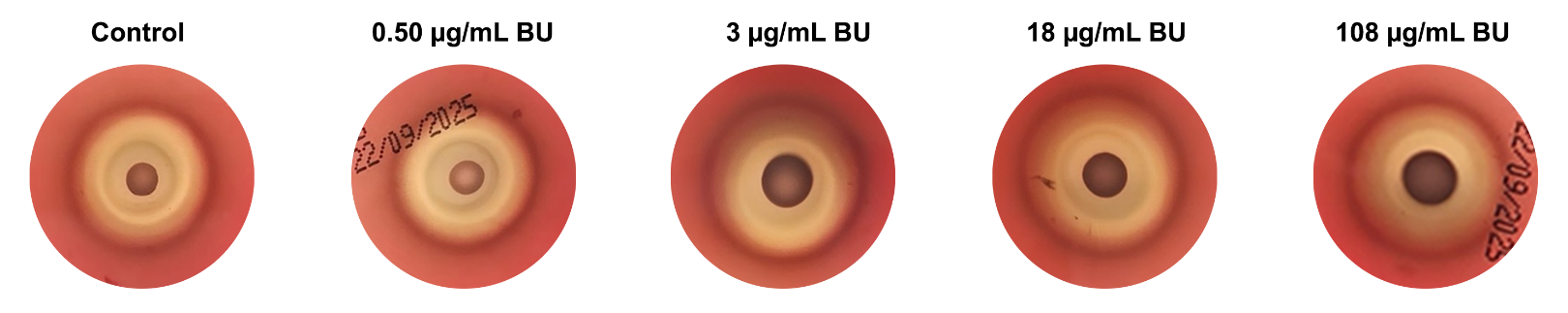
**

**Supplementary Table S1.** Receptor coordinates and box sizes and AutoDock VINA parameters.

| **Ligand & receptor** | **Receptor PDB ID** | **Co-crystalized ligand XYZ coordinates** | **Co-crystalized ligand radius (Å)** | **Set box size XYZ (Å)** | **Exhaust-iveness** | **Modes** | **Energy range (kcal/mol)** | **Grid space** |
| --- | --- | --- | --- | --- | --- | --- | --- | --- |
| **Bu-alg44** | 4RT0 | x: 24.042  y: 12.711  z: 93.065 | 12.50 | 30 | 128 | 40 | 15 | Default |
| **Bu-lasR** | 2UV0 | x: 23.016  y: 15.858  z: 80.066 | 8.34 | 22 | 128 | 40 | 15 | Default |
| **Bu-pqsE** | 5HIQ | x: −17.517  y: 2.435  z: −1.918 | 5.00 | 15 | 128 | 40 | 15 | Default |
| **Bu-qscR** | 6CC0 | x: −76.493  y: −9.502  z: 12.195 | 9.14 | 24 | 128 | 40 | 15 | Default |

**Supplementary Table S2.** Average OD600 reading and % inhibition of 6-fold of busulfan concentrations in µg/mL on *P. aeruginosa* strain PAO1.

| **Bu Concentration** | **WT** | |
| --- | --- | --- |
|  | **Avg. OD600** | **% of inhibition** |
| 0 µg/mL | 1.30 | 0 ± 0 |
| 0.5 µg/mL | 1.28 | 1.85 ± 2.7 |
| 3 µg/mL | 1.23 | 5.26 ± 4.7 |
| 18 µg/mL | 0.94 | 27.67 ± 3.25 |
| 108 µg/mL | 0.20 | 84.45 ± 8.23 |

**Supplementary Table S3.** Detailed molecular docking results using Vina.

| **Bu-receptor complex** | **Affinity (kcal/mol^-1^)** | **Total**  **H-bonds** | **Residue – Length (Interaction)** |
| --- | --- | --- | --- |
| **Bu-alg44** | –5.6 | 7 | SER88 – 2.68 (H-Bond)  ARG95 – 3.04 (H-Bond)  ARG21 – 2.52 (H-Bond)  ARG21 – 2.15 (H-Bond)  ARG21 – 1.96 (H-Bond)  ARG21 – 2.54 (H-Bond)  GLY97 – 3.65 (H-Bond)  PHE19 – 5.63 (Pi-Sulfur)  GLU97 – (vdW) |
| **Bu-lasR** | –6.4 | 4 | ARG61 – 2.80 (H-Bond)  ASP73 – 3.36(Carbon H-Bond)  THR75 – 3.54 (Carbon H-Bond)  PHE101 – 5.72 (Pi-Sulfur)  TRP60 – 5.82 (Pi-Sulfur)  ILE52 – (vdW)  GLY38 – (vdW)  LEU36 – (vdW)  TYP64 – (vdW)  TYR56 – (vdW)  LEU110 – (vdW)  TYR93 – (vdW)  THR115 – (vdW)  TRP – (vdW)  ALA127 – (vdW)  VAL76 – (vdW)  ALA70 – (vdW) |
| **Bu-pqsE** | –5.7 | 3 | ARG288 – 2.40 (H-Bond)  SER273 – 1.96 (H-Bond)  HIS282 – 2.76 (H-Bond)  HIS282 – 3.50 (H-Bond)  HIS71 – 4.06 (Pi-Sulfur) |
| **Bu-qscR** | –5.9 | 3 | SER38 – 1.86 (H-Bond)  SER129 – 1.90 (H-Bond)  TYR58 – 3.25 (H-Bond)  TYR58 – 5.73 (Pi-Sulfur)  ILE110 – (vdW) |

**Supplementary Table S4.** Summary of results conducted on PAO1 and Bu assays.

|  |  |  |  |  |  |  |
| --- | --- | --- | --- | --- | --- | --- |
|  |  |  | **Bu concentrations (µg/mL)** | | | |
|  | **Experiment** | **QC** | **0.5 µg/mL** | **3 µg/mL** | **18 µg/mL** | **108 µg/mL** |
|  | MIC % | 0 ± 0 | 1.85 ± 2.7 | 5.26 ± 4.7 | 27.67 ± 3.25**** | 84.45 ± 8.23**** |
|  |  |  |  |  |  |  |
| Biofilm | Biofilm formation % | 100 ± 30.3% | 45 ± 19.84% | 117.5 ± 28.39% | 142.5 ± 27.04% | 202.5 ± 27.04%** |
|  | Resazurin % | 100 ± 22.5% | 82.8 ± 13.5%* | 67.1 ± 6.7%**** | 54.2 ± 3.1%**** | 44.6 ± 0.07%**** |
|  | Live cells % | 81% | 60% | 51% | 43% | 33% |
|  | Dead cells % | 19% | 40% | 49% | 57% | 67% |
|  | Biomass of live cells (µm^3^/µm^2^) | 22.44 ± 8 µm^3^/ µm^2^ | 15.3 ± 3 µm^3^/ µm^2^** | 14.8 ± 2.9 µm^3^/ µm^2^*** | 9.26 ± 3.23 µm^3^/ µm^2^**** | 2.7 ± 0.9 µm^3^/ µm^2^**** |
|  | Biomass of dead cells (µm^3^/µm^2^) | 5.4 ± 2 µm^3^/ µm^2^ | 10.3 ± 3.2 µm^3^/ µm^2^* | 14.5 ± 4.7 µm^3^/ µm^2^*** | 11.95 ± 3.3 µm^3^/ µm^2^** | 5.5 ± 1.35 µm^3^/ µm^2^ |
|  | Biofilm thickness (µm) | 75 ± 15.26 µm | 49 ± 17.30 µm** | 46 ± 14.74 µm** | 36 ± 19.09 µm**** | 19 ± 12.78 µm**** |
|  |  |  |  |  |  |  |
| Pigments production (%) | Pyocyanin | 100 ± 7.1% | 125.3 ± 18%** | 135.7 ± 2.10%**** | 129.9 ± 17.5%*** | 42.27 ± 3.78%**** |
|  | Pyoverdine 12h | 100 ± 9.5% | 97.8 ± 4.9% | 102 ± 6.5% | 128.34 ± 19.5%** | 140.2 ± 17.17%**** |
|  | Pyoverdine 30h | 100 ± 10% | 85.64 ± 11.7% | 99.23 ± 3.5% | 95.7 ± 18.26% | 134.8 ± 3.3%**** |
| Motility (cm) | Swarming | 3.68 ± 0.07 cm | 3.65 ± 0.05 cm | 3.78 ± 0.04 cm | 3.86 ± 0.058 cm* | 3.71 ± 0.085 cm |
|  | Swimming | 6.91 ± 0.08 cm | 6.37 ± 0.115 cm | 6.7 ± 0.254 cm | 6.11 ± 0.42 cm** | 6.72 ± 0.092 cm |
|  | Twitching | 1.493 ± 0.19 cm | 1.84 ± 0.165 cm | 1.73 ± 0.093 cm | 2.023 ± 0.244 cm** | 1.60 ± 0.055 cm |
|  | Siderophore production (%) | 100 ± 1.8% | 96.47 ± 3.09% | 99.4 ± 2.09% | 95.4 ± 7.33% | 74.8 ± 19.7%** |
